# A Quantized CNN-Based Microfluidic Lensless-Sensing Mobile Blood-Acquisition and Analysis System

**DOI:** 10.3390/s19235103

**Published:** 2019-11-21

**Authors:** Yumin Liao, Ningmei Yu, Dian Tian, Shuaijun Li, Zhengpeng Li

**Affiliations:** School of Automation and Information Engineering, Xi’an University of Technology, Xi’an 710000, China; 1180311011@stu.xaut.edu.cn (Y.L.); 2180320059@stu.xaut.edu.cn (D.T.); 2180320068@stu.xaut.edu.cn (S.L.); 2180320070@stu.xaut.edu.cn (Z.L.)

**Keywords:** lensless sensing, quantization scheme, CNN, microfluidic chip, blood analysis

## Abstract

This paper proposes a microfluidic lensless-sensing mobile blood-acquisition and analysis system. For a better tradeoff between accuracy and hardware cost, an integer-only quantization algorithm is proposed. Compared with floating-point inference, the proposed quantization algorithm makes a tradeoff that enables miniaturization while maintaining high accuracy. The quantization algorithm allows the convolutional neural network (CNN) inference to be carried out using integer arithmetic and facilitates hardware implementation with area and power savings. A dual configuration register group structure is also proposed to reduce the interval idle time between every neural network layer in order to improve the CNN processing efficiency. We designed a CNN accelerator architecture for the integer-only quantization algorithm and the dual configuration register group and implemented them in field-programmable gate arrays (FPGA). A microfluidic chip and mobile lensless sensing cell image acquisition device were also developed, then combined with the CNN accelerator to build the mobile lensless microfluidic blood image-acquisition and analysis prototype system. We applied the cell segmentation and cell classification CNN in the system and the classification accuracy reached 98.44%. Compared with the floating-point method, the accuracy dropped by only 0.56%, but the area decreased by 45%. When the system is implemented with the maximum frequency of 100 MHz in the FPGA, a classification speed of 17.9 frames per second (fps) can be obtained. The results show that the quantized CNN microfluidic lensless-sensing blood-acquisition and analysis system fully meets the needs of current portable medical devices, and is conducive to promoting the transformation of artificial intelligence (AI)-based blood cell acquisition and analysis work from large servers to portable cell analysis devices, facilitating rapid early analysis of diseases.

## 1. Introduction

Currently, cell analysis plays an important role in the diagnosis and efficient evaluation of diseases, and the demand for mobile real-time detection of cells for personalized biomedical diagnosis will likely increase in the future [1]. However, with the traditional method, test samples must be prepared on glass slides and manual analysis by counting cells with a microscope is required. There are many problems with this, such as the large equipment volume, dependence on the operator’s professional knowledge, and the big difference in inspection results by different people [2]. The large equipment volume is inconvenient to move and expensive, preventing analysis anytime and anywhere. Furthermore, all analysis depends on professional operators, which is also costly and can easily lead to subjectivity among human assessors. Therefore, miniaturization and automation of blood analysis equipment is the expected development trend.

Cell analysis is very difficult to carry out if relying on the human eye, so it is almost impossible to undertake cell analysis by manual searching for image features. For this reason, artificial intelligence is a good solution. In 1998, Lecun designed the CNN named LeNet-5 [3] to recognize handwriting. In 2012, the recognition rate of AlexNet, which is a CNN model proposed by Krizhevsky, was greatly advanced [4]. However, most of the current CNN algorithms are not well suited for use on mobile devices, therefore CNN operations can only be run on high-performance servers, which also leads to the problem of expensive and immovable devices. At the same time, the traditional microscope acquisition system is also the main reason the equipment cannot be miniaturized. The lens and sample observation methods of the traditional microscope make it difficult to miniaturize the equipment. Therefore, it is also necessary to develop miniaturized blood image acquisition equipment different from the traditional microscope, in order to make the whole system miniaturized.

Living cells are very sensitive to temperature. In small integrated detection equipment, the ambient temperature can be increased easily due to circuit heating, and so the test results will be affected. A lower area means lower power and a lower equipment cost. For small microfluidic integrated detection equipment, a reduction in the circuit area can cause the temperature interference created by circuit heating to be greatly reduced. Therefore, our research focused on miniaturization by limiting the circuit hardware expense, and finding a good tradeoff between classification performance and computational expense, with the hope of improving the application of cell detection in remote clinics, telemedicine, and other fields.

We chose lensless image-sensing technology to solve the problem of miniaturization of acquisition equipment. In 2006, a lensless optical fluid microchip based on complementary metal oxide semiconductor (CMOS) image sensors and microfluidics the was first reported [5]. Over the past decade, lensless imaging technology has been developed rapidly with the improvement of the CMOS image sensor (CIS) and image-processing techniques [6,7,8,9,10,11,12]. Flow cytometry based on deep learning was also reported [13], which proposed a deep learning-based hologram reconstruction method. However, the inference process of deep learning still depends on a computer, which limits the further miniaturization of the system. At the same time, label-free bioaerosol sensing using mobile microscopy was reported [14], the proposed bioaerosol sensing and hologram reconstruction can achieve good results in image acquisition and classification. However, the bioaerosol-sensing system is not suitable for analysis outside a laboratory due to the need for a strict biosafety level laboratory.

Our research group has also undertaken a lot of research on lensless cell-detection systems [15,16]. Nowadays, most of the current CNN algorithms are not well suited for portable equipment. Following AlexNet [4], new CNN algorithms have primarily focused on the enhancement of classification accuracy. However, these CNN algorithms have not evolved a consideration of computing complexity and implementation efficiency. At the same time, with the rapid development of portable intelligent devices, the demand for small network models and limited hardware-cost CNN algorithms is increasing. This demand has led to a fast-developing research field that is focused on reducing the size of CNN models size and minimizing CNN accuracy losses.

One of the approaches for a possible solution is to quantize the CNN network parameters from floating-point 32 bit into low-precision representations. There are many approaches that could be used to achieve this, such as exclusive-not-or (XNOR) net [17], binary neural networks (BNNs [18]), and more [19,20,21,22,23,24]. However, the above quantization methods do not include consideration of efficiency improvements in hardware, because these approaches do not optimize the algorithm for the balance between hardware cost and accuracy. Some approaches only quantize the weights [25,26]. These approaches mainly consider the reduction of on-device storage, but neglect the hardware implementation efficiency. Bit-shift and binary networks [17,18] are exceptions, the weights of which are either 0 or powers of 2 in these two approaches. In this way, the bit-shift circuits can be implemented in multiplication. However, bit-shift only can bring a small benefit to the custom implementation of multiply-add calculations. Moreover, 1-bit quantization often leads to substantial performance degradation. A more meaningful attempt to quantize model architectures is required, which is already proven have a good performance in latency and accuracy.

In this paper, a mobile CNN lensless-sensing blood cell analysis hardware system was built to address the above issues. Our specific contributions are:

- A quantization algorithm for mobile hardware implementation, which supports different kernels with different quantization parameters, and has an optimal tradeoff between classification accuracy and hardware cost. (Section 2)
- A quantization circuit architecture for the quantization scheme. (Section 3)
- A dual register group structure to allow for pipelining of a quantized CNN architecture, thereby increase its throughput. (Section 4)
- A microfluidic chip and mobile lensless blood cell image acquisition device to build an entire mobile lensless microfluidic blood image acquisition and analysis system. (Section 5)
- Implementation of the quantization architecture in FPGA, and application of the cell segmentation and cell classification CNN in the system to demonstrate a blood cell segmentation and classification analysis task. (Section 6)
- The first miniaturization of a quantization CNN-based microfluidic lensless-sensing white blood cell (WBC) analysis system. This system has a significant tradeoff that enables miniaturization while retaining accuracy, and this promotes the research on mobile artificial intelligence (AI) diagnosis equipment.

## 2. Quantization Algorithm

This section describes our quantization algorithm, which is optimized for hardware implementation. In order to demonstrate the mapping relationship between the algorithm and the circuit more clearly, the whole algorithm description process is consistent with the data flow of the hardware in the next section.

### 2.1. Quantization Scheme

We used floating-point arithmetic in the CNN training, and integer arithmetic in the inference process, and they maintained a high degree of correspondence with each other.

The equivalent between the bit-representation of the value (denoted *q* below) and interpretation of the mathematical real value (denoted *r* below) is defined as:

(1) $$r_{d}=s_{d}(q_{d}+o_{d})2^{-n_{d}}$$

(2) $$r_{w}=s_{w}q_{w}2^{-n_{w}}$$

Equation (1) is the feature data quantization formula, and Equation (2) is the weight quantization formula, where suffix *d* denotes the feature data, and suffix *w* denotes weight.

In Equations (1) and (2), *r* denotes “real value”, *s* denotes “scale parameter”, *q* denotes “quantized value”, *o* denotes “offset parameter”, and *n* denotes “shift parameter”.

For X-bit quantization, *s*, *q* and *o* are quantized as an X-bit integer. For example, in 8bit quantization, *s*, *q* and *o* are quantized as an 8 bit integer.

The constant *s* (for “scale”) is an arbitrary positive real integer number. *s2^−n^* can typically be represented as a floating-point quantity.

### 2.2. Feature Quantization Parameter o_d_ s_d_ n_d_ Calculation

We now describe the calculation of the parameters *s*, *n* and *o*. The quantization methods are different for feature data quantization and weight quantization.

First, we obtained the maximum value (denoted *r_d_*_max_) and minimum value (denoted *r_d_*_min_) of the feature data range. If we take an 8 bit quantization as an example (int8), then the maximum and minimum values become 127 and −127, respectively. The corresponding quantized values, *q_d_*_max_ and *q_d_*_min_, can be expressed as the maximum and minimum value of feature data quantization.

(3) $$q_{d\max}=\frac{r_{d\max}2^{n_{d}}}{s_{d}}-o_{d}=127$$

(4) $$q_{d\min}=\frac{r_{d\min}2^{n_{d}}}{s_{d}}-o_{d}=-127$$

Subtracting Equation (3) from (4) results in:

(5) $$\frac{(r_{d\max}-r_{d\min})2^{n_{d}}}{s_{d}}=254$$

Rearranging Equation (5) then yields:

(6) $$s_{d}2^{-n_{d}}=\frac{r_{d\max}-r_{d\min}}{254}$$

From Equation (6), the parameter *s* and *n* for the feature data can be calculated. Thereafter, the resulting parameters *n* and s can be inserted into Equations (3) and (4) to compute the parameter *o*.

### 2.3. Weight Quantization Parameter s_w_ n_w_ Calculation

Our quantization algorithm uses different quantization parameters for different weight cube arrays.

Next, we describe the calculation of the parameters *s* and *n*. First, we found out the maximum value (denoted *r_w_*_max_) and minimum value (denoted *r_w_*_min_) in every weight cube array. If we take an 8 bit quantization as an example (int8), then the maximum and minimum values become 127 and −127, respectively. The corresponding quantized values, *q_w_*_max_ and *q_w_*_min_, can be expressed as the maximum and minimum value of weight quantization.

(7) $$q_{w\max}=\frac{r_{w\max}2^{n_{w}}}{s_{w}}=127$$

(8) $$q_{w\min}=\frac{r_{w\min}2^{n_{w}}}{s_{w}}=-127$$

Subtracting Equation (7) from (8) results in:

(9) $$\frac{(r_{w\max}-r_{w\min})2^{n_{w}}}{s_{w}}=254$$

Rearranging Equation (9) then yields:

(10) $$s_{w}2^{-n_{w}}=\frac{r_{w\max}-r_{w\min}}{254}$$

From Equation (10), the parameters *s* and *n* for the weight cube array can be calculated. Every weight cube array has its own quantization parameter.

### 2.4. Convolution Calculation

From the definition of the feature data quantization (1) and weight quantization (2) formulas, we have:

(11) $$q_{d}=\frac{r_{d}2^{n_{d}}}{s_{d}}-o_{d}$$

(12) $$q_{w}=\frac{r_{w}2^{n_{w}}}{s_{w}}$$

Then, the convolution MAC (multiply-accumulate) calculation becomes:

(13) $$\sum q_{w}q_{d}=\sum \frac{r_{w}r_{d}2^{n_{w}+n_{d}}}{s_{w}s_{d}}-\sum q_{w}o_{d}$$

In Equation (13), we denote the convolution results $\sum q_{w}q_{d}$ as *q_conv_*, and denote $\sum q_{w}o_{d}$ as *o_conv_*, and denote $\sum r_{w}r_{d}$ as *r_conv_*, and denote $\sum s_{w}s_{d}$ as *s_conv_*, and denote $n_{w}+n_{d}$ as *n_conv_*. Then, Equation (13) can be rewritten as:

(14) $$q_{conv}=\frac{r_{conv}2^{n_{conv}}}{s_{conv}}-o_{conv}$$

### 2.5. Unify Weight Cubes Convolution Result

Because our quantization algorithm uses different quantization parameters for different weight cube arrays, after convolution for each weight cube array, the convolution result of all weight cube arrays should unify to the same quantization parameter.

As we described above, the result of the convolution equation is Equation (14). Let the target quantization parameters be *s_tgt_*; *o_tgt_*; *n_tgt_*. The quantization equation is implemented here as:

(15) $$(q_{conv}+o_{conv})s_{conv}2^{-n_{conv}}=(q_{tgt}+o_{tgt})s_{tgt}2^{-n_{tgt}}$$

Then we have:

(16) $$q_{tgt}=\frac{(q_{conv}+o_{conv})s_{conv}2^{-n_{conv}}}{s_{tgt}2^{-n_{tgt}}}-o_{tgt}=(q_{conv}+o_{conv}-o_{tgt}\frac{s_{tgt}2^{-n_{tgt}}}{s_{conv}2^{-n_{conv}}})\frac{s_{conv}2^{-n_{conv}}}{s_{tgt}2^{-n_{tgt}}}$$

We use *s_chg_*; *o_chg_*; *n_chg_* to denote the quantization parameters for changing the current weight cube array convolution result to the target quantization parameter. From Equation (16), we can calculate the value of *s_uni_*; *o_uni_*; *n_uni_* as below.

(17) $$o_{uni}=o_{conv}-o_{tgt}\frac{s_{tgt}2^{-n_{tgt}}}{s_{conv}2^{-n_{conv}}}$$

(18) $$s_{uni}2^{-n_{uni}}=\frac{s_{conv}2^{-n_{conv}}}{s_{tgt}2^{-n_{tgt}}}$$

### 2.6. Bias Operation Quantization

For models which use biases, there is an addition operation. The addition operation in batch normalization is also implemented here.

To remove parameter o when the bias operation finishes, we add parameter *2^sft^* into the bias quantization formula.

(19) $$q_{b}=(\frac{r_{b}2^{n_{b}}}{s_{b}}-o_{b})2^{sft}$$

After unifying the weight cube array to target the quantization parameters, the bias addition equation is:

(20) $$q_{conv}+q_{b}=\frac{r_{conv}2^{n_{conv}}}{s_{conv}}-o_{conv}+(\frac{r_{b}2^{n_{b}}}{s_{b}}-o_{b})2^{sft}$$

The bias value is quantized as int8, in reference to the quantization method of the feature data.

(21) $$0<\;(q_{b\max}=\frac{r_{b\max}2^{n_{b}}}{s_{b}}-o_{b})<127$$

(22) $$-127<\;(q_{b\min}=\frac{r_{b\min}2^{n_{b}}}{s_{b}}-o_{b})<0$$

Subtracting Equation (21) from (22) results in:

(23) $$\frac{(r_{b\max}-r_{b\min})2^{n}}{s_{b}}<254$$

Then:

(24) $$s_{b}2^{-n_{b}}>\frac{r_{b\max}-r_{b\min}}{254}$$

Then let:

(25) $$\begin{array}{l} s_{conv}=s_{b}\\ n_{conv}=n_{b}+sft\\ n_{conv}+o_{b}2^{sft}=0 \end{array}$$

From Equations (20)–(25), the parameters *s_b_*; *n_b_*; *o_b_*; *sft* for bias can be calculated. Equation (20) then becomes:

(26) $$\begin{array}{l} q_{conv}+q_{b}\\ =\frac{r_{conv}2^{n_{conv}}}{s_{conv}}-o_{conv}+(\frac{r_{b}2^{n_{b}}}{s_{b}}-o_{b})2^{sft}\\ =\frac{r_{conv}2^{n_{conv}}}{s_{conv}}+\frac{r_{b}2^{n_{b}+sft}}{s_{b}}-o_{conv}-o_{b}2^{sft}\\ =\frac{r_{conv}+r_{b}}{s_{conv}}2^{n_{conv}} \end{array}$$

The result for the bias can then be written as:

(27) $$q_{bias}=\frac{r_{bias}}{s_{bias}}2^{n_{bias}}$$

The addition of the bias and the batch normalization eliminate the offset parameter *o*.

### 2.7. Batch-Normalization Multiplication Quantization

For models which use batch normalization [27], there is a multiplication operation.

Let *q_m_* denote the quantized multiplication value, and the multiplication operator quantization formula is:

(28) $$q_{m}=\frac{r_{m}2^{n_{m}}}{s_{m}}$$

Therefore, the multiplication equation is quantized as:

(29) $$q_{bias}\times q_{m}=\frac{r_{bias}}{s_{bias}}2^{n_{bias}}\times \frac{r_{m}2^{n_{m}}}{s_{m}}=\frac{r_{bias}r_{m}2^{n_{bias}+n_{m}}}{s_{bias}s_{m}}$$

The multiplication value also quantized as int8. From Equations (21), (22), (24) and (29), the parameter *s_m_*; *n_m_* for multiplication can be calculated.

Then, the result of batch normalization multiplication can be rewritten as:

(30) $$q_{bn}=\frac{r_{bn}}{s_{bn}}2^{n_{bn}}$$

### 2.8. Layer Output Quantization

After calculating per layer, the bit width of the resulting value is more than 8 bit, and the output per layer will be the next layer’s input data. Therefore, the calculation result needs to be quantized into int8 before being output.

Let *q_out_* denote the quantized output value, and the output value quantization formula is:

(31) $$q_{out}=(q-o_{out})s_{out}2^{-n_{out}}$$

Because the *q* after bias and batch normalization is:

(32) $$q=\frac{r}{s}2^{n}$$

Inserting Equation (32) into (31) results in:

(33) $$q_{out}=(\frac{r\times 2^{n}}{s}-o_{out})\times s_{out}\times 2^{-n_{out}}=(r-\frac{o_{out}s}{2^{n}})\frac{s_{out}}{s}\times 2^{n-n_{out}}$$

Like the quantization of the feature data in Section 2.2, let

(34) $$q_{out\max}=\frac{r_{out\max}2^{n_{d}}}{s_{out}}-o_{out}=127$$

(35) $$q_{out\min}=\frac{r_{out\min}2^{n_{d}}}{s_{out}}-o_{out}=-127$$

Subtracting Equation (34) from (35) gives:

(36) $$s_{out}2^{-n_{out}}=\frac{r_{out\max}-r_{out\min}}{254}$$

The output value is quantized as int8. Then, in reference to the quantization method of the feature data, from Equations (33)–(36), the parameters *s_out_*; *n_out_*; *o_out_* for the output quantization can be calculated.

## 3. Quantization CNN Circuit Architecture

**Figure 1:**
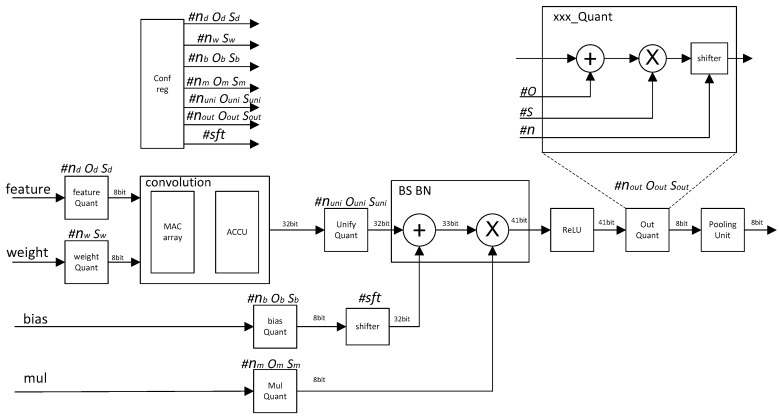
Integer quantization CNN accelerator architecture diagram.

We propose a quantization CNN accelerator circuit architecture for the above quantization scheme. The architecture is shown in Figure 1.

All the quantization parameters are calculated offline. The quantization parameters include *o_x_ n_x_ s_x_* in each phase, and *sft* values for the shifter module. The calculation method of each quantization parameter is described in detail in the previous algorithm introduction section.

**Figure 2:**
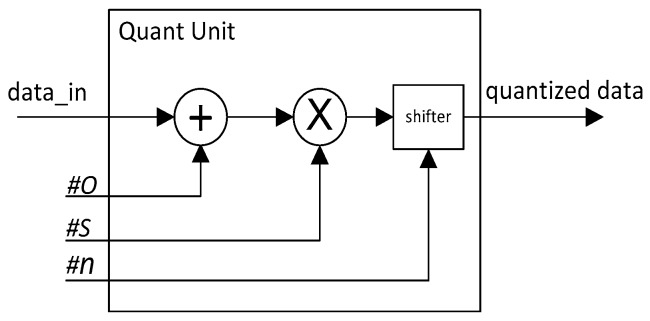
Quantization operation unit structure diagram.

According to our proposed quantization Equations (1) and (2), the quantization operation is easy to implement in hardware. The structure of the quantization operation unit is shown in Figure 2.

The workflow of the quantized CNN acceleration circuit is as follows.

The quantization parameters of all modules were stored in the configuration register unit.

After the Feature_data and weight were input, the feature_quantizatier and weight_quantizatier quantized the feature data and weight into 8 bit.

The MAC_array and accumulation unit (ACCU) completed the convolution operation and the result became 32 bit. Since the quantization parameters of each kernel cube array were different, after each kernel convolution operation was completed, all the different quantization parameters of all kernel cube arrays needed to be unified by Unify_quantizatier.

The bias and bn operations were completed by the quantize adder and multiplier, and the value became 41 bit. Then, the activation operation was completed through the ReLU unit.

The value from the ReLU unit was quantized to 8 bit by the output quantization unit, and then the pooling operation was completed by the pooling_unit.

## 4. Dual Register Group Structure

**Figure 3:**
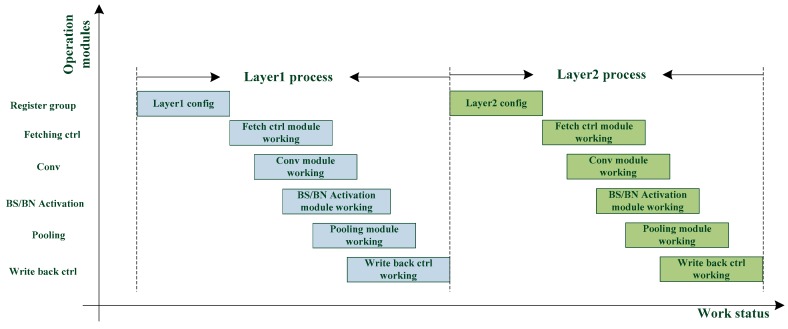
Processing of a classical CNN accelerator.

The classical neural network acceleration operation flow is that a layer requires the computational results of its preceding layer. This dependency is illustrated in Figure 3, indicating how substantial time is lost when waiting for the computation of a layer to finish.

**Figure 4:**
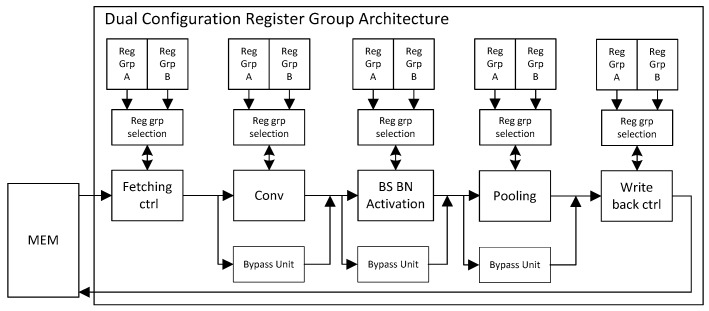
Dual configuration register group structure diagram.

The use of a circuit without a pipeline structure has a low throughput rate. For this case, we propose a dual-register pipeline circuit structure, which can further enhance the calculation efficiency of the acceleration circuit. Every module has its two register groups (Reg_Grp_A and Reg_Grp_B) and register group selection unit (RSU), which is primarily used to store the network parameter configuration of the current layer and next layer. The structure is shown in Figure 4.

**Figure 5:**
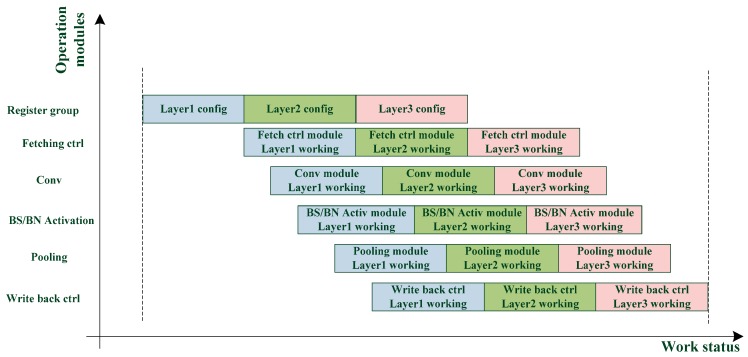
Processing of dual configuration register group structure.

The dual register group structure allows each module to perform the next layer immediately after the current layer calculation. As shown in Figure 5, the waiting time of each module is greatly reduced, and the calculation efficiency is improved.

A three-layer network is taken as an example to illustrate the working flow of the circuit:

(1) Configure the parameters of the first layer and the second layer into Reg_Grp_A and Reg_Grp_B, respectively.
(2) The parameters in Reg_Grp_A are applied to perform the current layer operation, and a completion signal will be sent to RSU when the operation is accomplished.
(3) After the RSU receives the first layer operation completion signal, the parameters in the Reg_Grp_B are loaded for the second layer calculation and the parameters of the third layer are configured into the Reg_Grp_A.
(4) When the operation of the second layer is completed, the operation completion signal is also sent to the RSU again.
(5) After the RSU receives the second layer operation completion signal, the parameters in the Reg_Grp_A are loaded for the third layer calculation.
(6) After the calculation of the third layer is completed, it is also the end of three-layer network calculation.

In this structure, each module is pipelined, which improves the computational efficiency of the CNN accelerator.

## 5. Cell Segmentation and Classification CNN

In medical image analysis, cell segmentation is one of the most basic and important research tasks. It is also the basic premise of cell image recognition and counting. In our previous studies, we proposed two algorithms: blood cell image-segmentation based on a convolutional neural network [28], and the classification of white blood cells by CNN [29]. We applied both to the accelerator, and introduce them in this section.

Pixel size is one of the key factors affecting image quality in lensless imaging systems. The resolution of the cell image captured by the lensless system is lower than that captured by an optical microscope. Moreover, the noise is larger and the boundary information is more ambiguous. Noise-sensitive image thresholding and cell segmentation methods are not suitable for this scenario.

**Figure 6:**
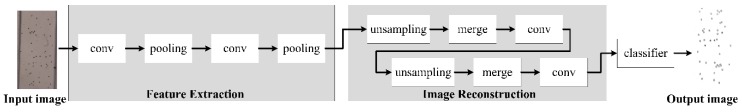
Cell Division Network (CSnet) structure.

In order to addresses these issues, we have optimized the Unet [30] model. The optimized model is called CSnet (Cell Division Network) as shown in Figure 6. According to the imaging characteristics of a lensless imaging system, a series of convolution output features are compensated in time. The CSnet network structure also solves the problems of the long running time and high storage space requirement of the training system, which are not conducive to porting applications.

**Figure 7:**
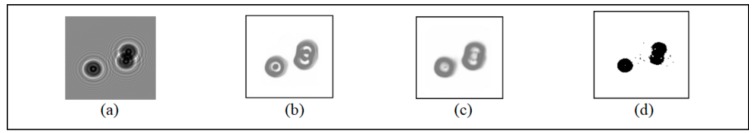
Comparison results: (**a**) original image (**b**) result of CSnet (**c**) result of Unet (**d**) result of the self-adaptive threshold algorithm.

We compared CSnet, Unet and the adaptive threshold segmentation algorithm, and the results are shown in Figure 7.

In Table 1, four evaluation criteria (Jaccard, confirm-index, precision and recall) are applied to quantify the results of segmentation. In the field of image segmentation, a higher score means a better effect of segmentation, and a score of 1 means perfect consistency in the segmentation. Unet and CSnet are compared with these four evaluation criteria in Table 1, and the better result was obtained by CSnet.

**Figure 8:**
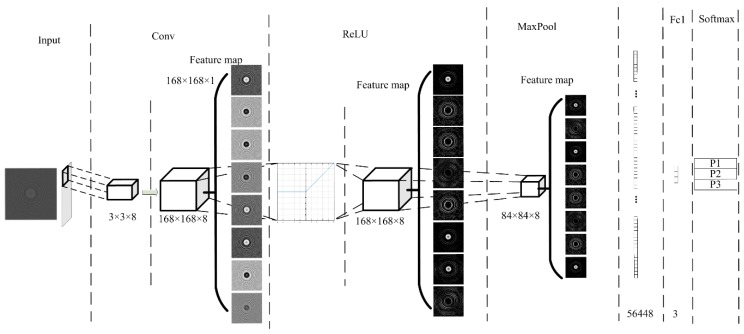
White blood cell (WBC) classification network structure.

White blood cells (WBCs) are the nucleated cells in peripheral blood. Their quantity is only 0.1~0.2% that of red blood cells (RBCs). At present, the most advanced algorithm in image classification is CNN, and this method fully meets the application requirements for detecting WBCs. Therefore, we also employed our proposed CNN architecture to classify WBCs. The specific network structure is shown in Figure 8.

We used the LeNet-5 structure to design the algorithm. The input image of the input layer was processed by the proposed cell segmentation algorithm. In the convolution layer, the size of the convolution kernel was 3 × 3 × 8 and the step size was 1. The network structure had only one layer of convolution. Then, the ReLU activation function was used to improve the classification accuracy. The maximum pooling method was adopted, with a pooling window of 2 × 2 and a step size of 2. After the first pooling, the feature map size was 84 × 84 × 8, so the full connection layer size was 56,448. The output size was 3, which represented three kinds of WBCs: lymphocytes, mononuclear, and neutrophils. The probability of the three kinds of WBCs was calculated by using the soft Max function.

## 6. Mobile Microfluidic Acquisition Device

This section introduces the proposed/designed microfluidic acquisition device. The microfluidic acquisition device consists of two parts: the microfluidic chip and the lensless image-sensing module. The system operates as follows:

(1) Place a microfluidic chip in the lensless image sensing device.
(2) A group of micropumps is used to control the flow rate to ensure that the lensless imaging device can acquire appropriate images.
(3) The detected samples are injected into the microfluidic chip, and the data are collected by the lensless image sensing module.

### 6.1. Process Flow of the Microfluidic Chip

**Figure 9:**
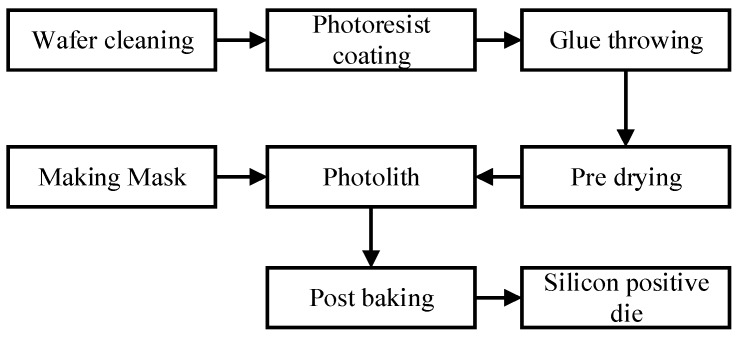
Processing of the silicon wafer positive die.

The fabrication of the microfluidic chip can be divided into two parts: the fabrication of silicon cathode chips and the fabrication of microfluidic chips. The process of silicon positive die is shown in Figure 9. First, we designed the required microchannel graphics and made the mask. Then the silicon wafers were cleaned by the standard cleaning process and the photoresist was coated. Finally, the masks and silicon wafers coated with photoresist were used for photolithography and development, and the preparation of the positive die was completed. We used type SU-8 photoresist. This photoresist ensures that a highly suitable microfluidic chip channel can be fabricated.

**Figure 10:**
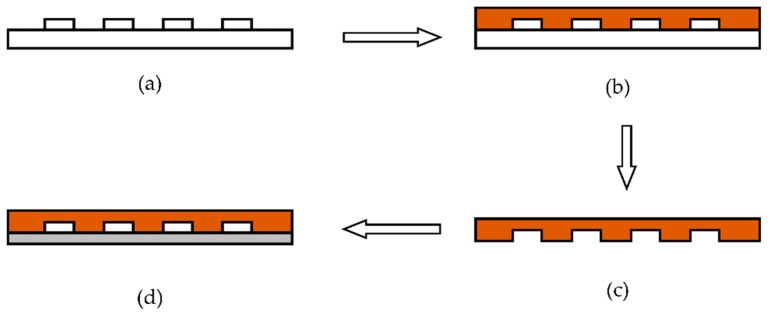
Fabrication of the PDMS microfluidic chip by the pouring process. (**a**) Surface modification on positive die. (**b**) Pouring PDMS without bubbles on to the positive die. (**c**) Curing and separation. (**d**) Bonding glass to PDMS.

After the fabrication of the silicon positive die, the microfluidic chip was fabricated by the process shown in Figure 10. The whole process can be divided into the following four steps.

(1) Using trimethylchlorosilane to perform surface modification on the positive die.
(2) Pouring polydimethylsiloxane (PDMS) without bubbles onto the positive die.
(3) The positive die from step 2 is put into a baking oven for 0.5 h to cure, then the positive die and PDMS are stripped.
(4) Cleaning the thin glass and bonding it to PDMS.

**Figure 11:**
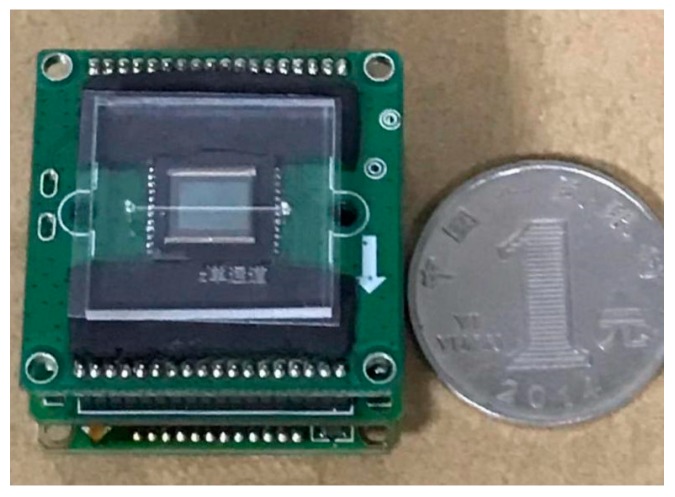
Comparison between the microfluidic chip and a renminbi (RMB) coin.

The fabricated microfluidic chip is shown in Figure 11.

### 6.2. Design of the Lensless Image-Sensing Module

The lensless image sensing module mainly consists of a light source and lensless-imaging sensor. The light source for the lensless imaging technology can be divided into the point light source and surface light source. The point light source has strong diffraction and is suitable for scenes requiring diffraction recovery. Small diffraction and clear imaging are characteristics of the surface light source. Considering the arithmetic operation and imaging quality, the surface light source was chosen as the system light source. The commercial OV5640 sensor was selected as the image sensor. The sensor had a pixel size of 1.4 um and a photosensitive area of 3673.6 um × 2738.4 um.

**Figure 12:**
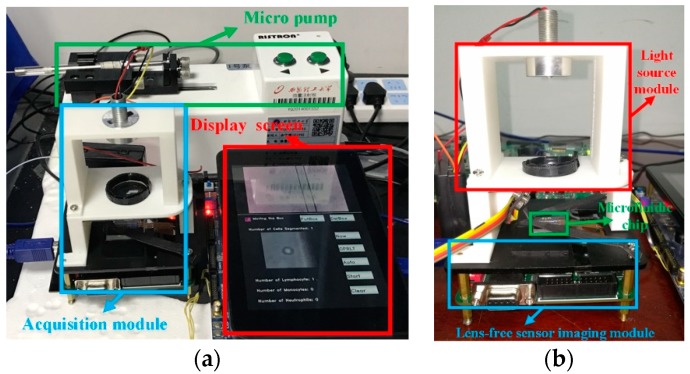
The microfluidic acquisition device (**a**) vertical view (**b**) side view.

We used a 0.1 mm precision 3D printer to complete the production of the system. The final microfluidic acquisition device is shown in Figure 12.

## 7. Experimental Results and Discussion

**Figure 13:**
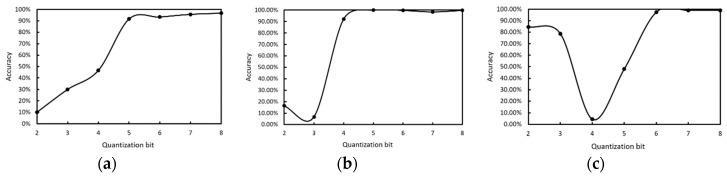
The accuracy of different quantization bits in the WBC classification network. (**a**) lymphocytes, (**b**) monocytes, (**c**) neutrophilic.

To verify the effect of the proposed integer quantization algorithm, we used different quantization bits in WBC classification network inference, and the quantization bit was from int2 to int8. The classification results of three kinds of white blood cells are shown in Figure 13. The more quantized the bits, the higher the accuracy, and 8 bit quantization is close to saturation.

Then, we compared the accuracy of different quantization bits with the floating point in the WBC classification network. The result is shown in Table 2. As expected, 8 bit integer quantization can achieve 98.44% accuracy, which is similar to 32 bit floating-point; the reduction in accuracy is only 0.56%.

To verify the benefits of using the integer quantization structure for the CNN accelerator hardware circuit, we also designed fp16 and int16 precision circuits with the same parallelism as our int8 quantization circuit. We used Synopsys design compiler (DC) to synthesize three kinds of quantization circuit, and the results of circuit area comparison are shown in Table 3. It shows that quantization greatly saves the area of hardware circuits, and the improvement is significant. We defined the figure of merit (FoM) as below:

FoM = 1/(accuracy_drop × area_percent)(37)

For example, int16 FoM is 1/(0.49 × 0.6702) = 3.045. The comparison result is shown in Table 3. As a result, 8 bit integer quantization has the best FoM of 3.239, in which the accuracy reduction is only 0.56%, and the area reduction is 44.86%.

To verify the effect of the dual configuration register group, we carried out experiments on the WBC classification network and compared the operation time of the dual-reg mode and common mode. The frequency of the operation clock was 100 MHz. The simulation operation time results are shown in Table 4. As expected, the dual-reg mode greatly increases the overlap operation time between CNN layers, thereby reducing the total operation time by 28.25%.

Then we implemented the CNN accelerator in the FPGA, and the FPGA was Xilinx Zynq UltraScale+ ZCU102. The maximum frequency was 100 MHz, Table 5 shows the experimental results of our int8 quantization and other previous quantization works [31,32]. Compared with the int8 quantization method [32], our int8 quantization method gives an increment of 0.67% on classification accuracy, and the hardware resource consumptions of look up table (LUT), flip-flop (FF), digital signal processor (DSP) and block random access memory (BRAM) were reduced by 18.54%, 24.87%, 71.07%, and 29.25%, respectively. Therefore, implementing our method helps to achieve a smaller hardware cost and better tradeoff between accuracy and hardware cost.

**Figure 14:**
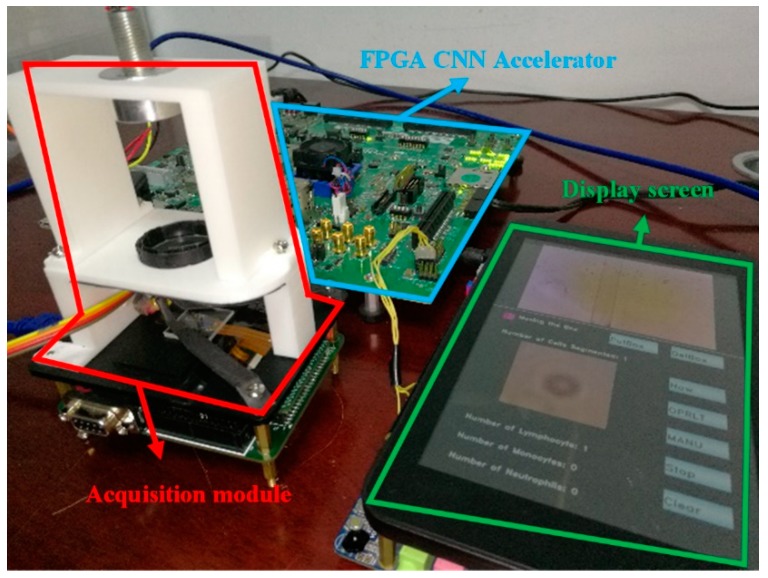
Mobile CNN microfluidic blood-acquisition and analysis prototype system.

We combined the FPGA CNN accelerator and the cell acquisition system to establish a complete CNN-based mobile lensless microfluidic blood acquisition and analysis prototype system, which is shown in Figure 14.

**Figure 15:**
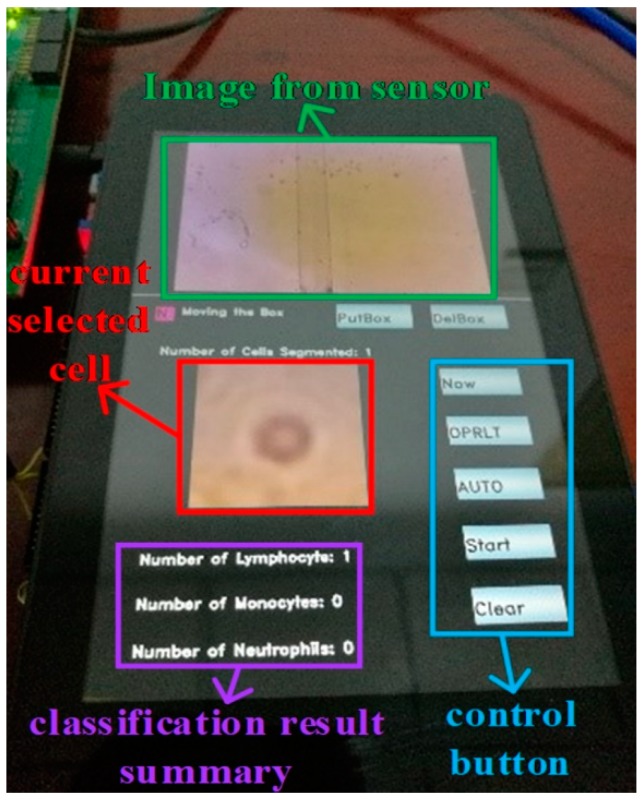
Touch panel display illustration.

Then we used our segmentation network to segment the blood images collected by the lensless microfluidic chip acquisition module. After the segmentation, we used the WBC classification network to classify the WBCs one by one. Finally, the classification results are displayed by statistics. The illustration of the touch panel is shown in Figure 15.

The experiments showed that the system can acquire, segment and classify cell images correctly. When the FPGA frequency is 100 MHz, a classification speed of 17.9 fps can be obtained, and the accuracy is 98.44%. Compared with the floating-point method, the accuracy dropped by only 0.56%, but the area decreased by 45%.

## 8. Conclusions

This paper proposed a CNN-based mobile microfluidic lensless sensing blood acquisition and analysis system, and an integer-only quantization scheme is proposed and applied to this system. Compared to floating-point based detection, a better tradeoff between accuracy and hardware cost was achieved. The CNN microfluidic lensless-sensing blood-acquisition and analysis system fully meets the needs of current portable medical devices and is conducive to promoting the transformation of AI based cell acquisition and analysis work from large servers to portable cell analysis devices, facilitating rapid early analysis of diseases. With the proposed quantization and its hardware implementation, it was thus illustrated that a miniaturization of the system is indeed viable. An accuracy of 98.44% with 17.9 fps rivals that of bulky in-clinic systems and thus paves the way for miniaturization of such systems.

## Figures and Tables

**Table 1:** Comparation of algorithm results.

Algorithm	Evaluation Standard of Image Segmentation			
	Jaccard	Confirm_Index	Precision	Recall
ostu	0.9161	0.9084	0.9465	0.9494
Unet	0.9245	0.9099	0.9551	0.9881
CSnet	0.9465	0.9434	0.9631	1

**Table 2:** Precision of the integer-quantized WBC classification network compared with floating point.

Precision	Float32	Int8	Int7	Int6	Int5	Int4
Accuracy	99.00%	98.44%	97.67%	96.68%	78.89%	47.67%
Accuracy Drop	-	–0.56%	–1.33%	–2.22%	–19.11%	–51.33%

**Table 3:** Accuracy and area comparison of different precision.

Precision	Float32	Int16	Int8	Int7	Int6	Int5	Int4
Accuracy	99.00%	98.51%	98.44%	97.67%	96.68%	78.89%	47.67%
Accuracy Drop	-	−0.49%	−0.56%	−1.33%	−2.22%	−19.11%	−51.33%
Area Drop	-	−32.98%	−44.86%	−45.07	−47.12	−49.09	−50.81%
FoM		3.045	3.239	1.376	0.852	0.103	0.039

**Table 4:** Operation efficiency improvement of the dual register group structure in the WBC classification network.

	Config	Data Fetch	Calculation	Overlap	Common Mode Summary	Dual-Reg Mode Summary	Operation Time Saved
Operation time (us)	1342	10,977	3949	4596	16,268	11,672	28.25%

**Table 5:** Experimental results of our int8 quantization and other previous quantization works.

	[31]	[32]	[32]	Ours
Format	32 bit float	32 bit float	8 bit integer	8 bit integer
Frequency	100 MHz	100 MHz	100 MHz	100 MHz
LUT	55,745	36,725	19,753	16,090
FF	45,561	37,283	20,938	15,731
DSP	-	220	121	35
BRAM	150.5	150	73.5	52
Classification Accuracy	98.18%	98.76%	97.77%	98.44%
